# Association of Gut Microbiome and Vitamin D Deficiency in Knee Osteoarthritis Patients: A Pilot Study

**DOI:** 10.3390/nu13041272

**Published:** 2021-04-13

**Authors:** Boopalan Ramasamy, Fabien Magne, Sujit Kumar Tripathy, Giriprasad Venugopal, Diptasree Mukherjee, Ramadass Balamurugan

**Affiliations:** 1 Department of Orthopaedics, Christian Medical College, Vellore, Tamil Nadu 632004, India; jpboopy@gmail.com; 2 Microbiology and Mycology Program, Biomedical Sciences Institute (ICBM), School of Medicine, University of Chile, Santiago Región Metropolitana 8380418, Chile; fabienmagne@med.uchile.cl; 3 Department of Orthopaedics, All India Institute of Medical Sciences, Bhubaneswar 110608, India; ortho_sujit@aiimsbhubaneswar.edu.in; 4 Center of Excellence for Clinical Microbiome Research, All India Institute of Medical Sciences, Bhubaneswar 110608, India; giriprsd36@gmail.com; 5 Department of Biochemistry, All India Institute of Medical Sciences, Bhubaneswar 110608, India; dr.diptasree@gmail.com

**Keywords:** knee osteoarthritis, vitamin D, gut microbiome

## Abstract

Background: Few preclinical studies have shown that Knee osteoarthritis (KOA) is linked to gut microbiome dysbiosis and chronic inflammation. This pilot study was designed to look at the gut microbiome composition in KOA patients and normal individuals with or without vitamin D deficiency (VDD, serum vitamin D <30 ng/mL). Methods: This pilot study was conducted prospectively in 24 participants. The faecal samples of all the participants were taken for DNA extraction. The V3-V4 region of 16s rRNA was amplified, and the library was prepared and sequenced on the Illumina Miseq platform. Results: The mean (±SD) age was 45.5 (±10.2) years with no defined comorbidities. Of 447 total Operational Taxonomic Units (OTUs), a differential abundance of 16 nominally significant OTUs between the groups was observed. Linear discriminate analysis (LEfSe) revealed a significant difference in bacteria among the study groups. Pseudobutyrivibrio and Odoribacter were specific for VDD, while Parabacteroides, Butyricimonas and Gordonibacter were abundant in the KOA_VDD group, and Peptococcus, Intestimonas, Delftia and Oribacterium were abundant in the KOA group. About 80% of bacterial species were common among different groups and hence labelled as core bacterial species. However, the core microbiome of KOA and VDD groups were not seen in the KOA_VDD group, suggesting that these bacterial groups were affected by the interaction of the KOA and VDD factors. Conclusion: Parabacteroides, Butyricimonas, Pseudobutyrivibrio, Odoribacter and Gordonibacter are the predominant bacteria in vitamin D deficient patients with or without KOA. Together these results indicate an association between the gut microbiome, vitamin D and knee osteoarthritis.

## 1. Introduction

Osteoarthritis (OA) of the knee is a degenerative joint condition that leads to progressive deterioration of the articular cartilage, subchondral bone sclerosis and synovial inflammation, resulting in joint pain and disability [1]. There is no definite treatment for this condition. The patients are treated with analgesic, calcium, vitamin D or focal physical interventions for symptomatic relief during the early stages [2,3,4,5,6]. In the advanced stage, joint replacement remains the only option that incurs several physical restrictions. Besides this, the surgical option is not durable. Consequently, researchers are trying to understand the disease aetiology so that it can be prevented or the course of disease can be modified. While multiple factors such as age, sex, ethnicity, obesity, vitamin D deficiency and genetic associations have been considered central contributing factors associated with disease progression [7,8,9,10], this disease’s aetiology remains a “mystery”. Although obesity increases OA risk through increased mechanical loading on the weight-bearing joints, it has been shown to increase the risk for OA even in the non-weight-bearing joints as well [11,12], suggesting that it might be caused by a low-grade systemic inflammation [13,14].

Besides, various studies show that the gastrointestinal microbiome contributes to a triggering of this low-grade systemic inflammation in obesity [14,15], through the bacterial endotoxin lipopolysaccharide (LPS) [16]. Moreover, LPS has been retrieved at an elevated concentration in the blood and the synovium of OA patients. It is also associated with severe knee OA, knee pain, and inflammation [17]. Other studies have also associated the gut-microbiome composition with a low-grade systemic and local inflammation observed in OA [18,19,20]. Together these findings support that the intestinal microbiome contributes to OA pathogenesis, although this interaction has still not been defined and needs to be further explored. Few recent studies reported the association of physical exercise to OA [20,21]. Physical activity could modulate the gut microbiome composition, boosting intestinal mucosal immunity, increasing the Bacteroidetes-Firmicutes ratio, modifying the bile acid profile and improving the production of short-chain fatty acids. Thus, physical exercise helps in the prevention and treatment of OA [20,21].

On the other hand, observational studies found that low levels of vitamin D were associated with a higher prevalence of KOA, along with an increased risk of disease progression [22,23,24]. In effect, vitamin D could be considered an essential factor in OA pathogenesis since vitamin D was shown to reduce cartilage degradation, which characterizes osteoarthritis [25,26]. So, it appears that vitamin D insufficiency or deficiency is closely associated with OA and its associated health implications [27]. Dietary nutrients shape the gut microbiome, and Vitamin D supplementation influences the microbiome [28]. Remarkably, vitamin D improves barrier function in the gut by inducing the expression of E-cadherin and improving the epithelial cell junctions in the gut [27]. Vitamin D deficiency can cause a breakdown of these epithelial cell junctions resulting in a leaky gut [16]. It affects the composition of the intestinal microbiome mainly through the expression of the cathelicidin antimicrobial peptide (CAMP) gene [29]. So, the dysbiosis induced by vitamin D deficiency might contribute to the severity of the OA by increasing intestinal barrier permeability and then producing LPS-mediated intestinal inflammation. We hypothesize that vitamin D deficiency and KOA may be associated with distinct microbiome signatures. We conducted a clinical pilot study to compare the gut microbial composition of KOA patients with or without vitamin D deficiency with that of normal individuals with low and high serum vitamin D levels to investigate this interrelation between vitamin D deficiency, microbiome and KOA.

## 2. Material and Methods

### 2.1. Study Participants

This observational study was conducted in a tertiary care teaching hospital between June 2015 and December 2016 to investigate the association of gut microbiome and serum vitamin D status in knee osteoarthritis (KOA) patients and in normal healthy individuals. The diagnosis of KOA was based on the American College of Rheumatology Criteria. All patients of primary KOA between 30 to 60 years of age and willing to participate in the study with a follow up of six months were included. Patients excluded were those with varus deformity > 5 degrees, valgus deformity, flexion deformity > 5 degrees, on anticoagulants, BMI > 30 kg/m^2^, history of hospitalization in last eight weeks, on antibiotics for last 12 weeks, gastrointestinal comorbidities (including inflammatory bowel disease, irritable bowel syndrome, gastrointestinal malignancies, surgical resection) or on pharmacologic doses (>3× recommended daily allowance) of vitamins or minerals were excluded. The Institutional Ethics Committee clearance was obtained (IRB Min No: 9432-29/04/2015), and the patients were recruited after getting their written informed consent. The KOA patients were categorized into one of the following two groups; Group 1 with vitamin D levels <30 ng/mL were classified as vitamin D deficient (KOA_VDD, *n* = 7), and Group 2 with vitamin D levels > 30 ng/mL were classified as normal vitamin D status (KOA, *n* = 4). The asymptomatic individuals (patient attendant or hospital staff) were categorized into Group 3 (vitamin D levels < 30 ng/mL, VDD, *n* = 7) and Group 4 (vitamin D levels > 30 ng/mL, normal/NVD, *n* = 6). There were no differences in age, geography and gender between the four groups.

### 2.2. Data/Specimen Collection

The demographic profile, socioeconomic status and medical history were entered into a predesigned proforma. The dietary intakes of food groups and macronutrients were calculated from 24 h dietary recall and food frequency. The daily ingestion of macro and micronutrients were matched with recommended dietary allowances for Indians using the DIGEST programme by ICMR [30]. The KOA disability was assessed using the WOMAC score [31].

For KOA_VDD and KOA groups, plain radiograph of both Knees (Anteroposterior and lateral views) in standing position were taken. The severity of KOA was classified as per Kellgren-Lawrence grade. Blood samples were collected in all four groups, and the following parameters were checked: serum haemoglobin level, erythrocyte sedimentation rate, C-reactive protein, serum creatinine, serum uric acid, serum calcium/phosphate, serum alkaline phosphatase, parathyroid hormone and thyroid function test and vitamin D levels. The faecal samples were collected from all participants in a plastic container on ice and then aliquoted in the lab, within 2 h, into a 2 mL Eppendorf tube for storage at −80 °C.

## 3. DNA Isolation, V3–V4 Region 16 s rRNA Sequencing and Library Preparation

A 200 mg of faecal sample from the Eppendorf tube was taken for DNA isolation with a modified DNeasy PowerLyzer Power Soil kit (Cat No: 12855-100, Qiagen, Qiagen GmbH, Hilden, Germany). The eluted DNA was quantitated using Nanodrop and checked using 1% agarose gel electrophoresis. The extracted faecal DNA was used to amplify the V3-V4 region of the 16s rRNA gene using region-specific primers (Table 1) with Illumina sequencing adapter overhangs and index barcode. PCR mix included 5ng of DNA to enrich 16s rRNA V3-V4 region and library preparation. The PCR reaction contained 25 pmol of each primer, 0.3 mM of each dNTP, 1.5 mM MgCl_2_ and 1U of High-Fidelity DNA polymerase (KAPA Biosystems, USA). The PCR amplification involved initial denaturation at 95 °C for 5 min followed by 1 min each 95 °C, 50 °C and 72 °C for 25 cycles and with a final extension at 72 °C for 7 min. The PCR amplicons were purified using Ampure XP beads (Beckman Coulter, USA) by following the manufacturer’s instructions. The amplicon Library Quality control was checked based on the size of the amplicon using Agilent Bioanalyzer DNA 7500 chip. All the libraries showed an expected size range (~600), indicating V3–V4 region amplification. The QC passed libraries was pooled in an equimolar concentration. The pooled libraries were sequenced using Illumina Miseq v2 Paired-End Reagents and Miseq instrument to generate 250 bp paired reads. FastQC and Rqc were used to check the quality parameters of sequence data like base call quality distribution, % bases above Q30, GC% sequencing adapter contamination etc. [32].

## 4. Data Analysis

For downstream analysis of the metagenome data, “Mothur” software bundle was used. The quality-filtered sequence reads were imported into Mothur, and the read pairs were aligned with each other with a minimum of 30 bp overlap to form contigs. Reads with ambiguous base calls were rejected. The high-quality contigs were checked for identical sequences, and duplicates were merged. The quality-filtered contigs were classified into taxonomical outlines based on the Silva NR v123 database. The classified contigs were filtered for any undesirable lineage like Mitochondria, Chloroplast, Fungi or Archaea. The contigs were then clustered into Operational Taxonomic Unit (OTUs) based on the phylotype. The abundance of each OTU in the samples was estimated. The alpha and beta diversity of OTUs between the groups was tested by the Kruskal–Wallis test after normalizing the OTU abundance. The relative abundance of bacterial groups was analyzed using Linear discriminant analysis effect size (LEfSe) [33]. Canonical correspondence analysis (CCA) was performed using R version 3.1.0 on a Bray–Curtis dissimilarity matrix. Adonis was used to test the association between the distinct groups and the overall microbiota composition based on distance matrices using the “Adonis” function in the “vegan” R package.

## 5. Results

A total of 24 participants, aged 45.5 ± 10.2 (mean ± SD) years, were recruited into four groups, with seven, four, seven and six participants in KOA_VDD, KOA, VDD and Normal (NVD) groups respectively (Table 2). These participants were predominantly rural or semi-urban dwellers belonging to middle- to lower-middle socioeconomic status. None of them was on vitamin D supplementation, and they did not consume milk. They had no other comorbidities. The mean BMI was >30, with the highest BMI 28.9 ± 2.6 (mean ± SD) in the VDD group and the lowest BMI 22.9 ± 2.5 (mean ± SD) in the Normal group. The normal group was significantly different (*p* < 0.05) from all the three-study group; however, BMI among the three-study groups was not different. Their haemoglobin levels were 13 ± 1.7 (mean ± SD) gm/dl, and none of them was on any treatment. KOA and KOA_VDD groups had a chronic history of pain ranging from four weeks to three years, and they performed only mild to moderate physical activity and had a WOMAC score of 57.6 ± 15.1 and 54.3 ± 10.7 (mean ± SD), respectively. All participants in study groups KOA_VDD, KOA and VDD had an occupation that kept them indoors.

## 6. Gut Microbiome Profile

**Figure 1:**
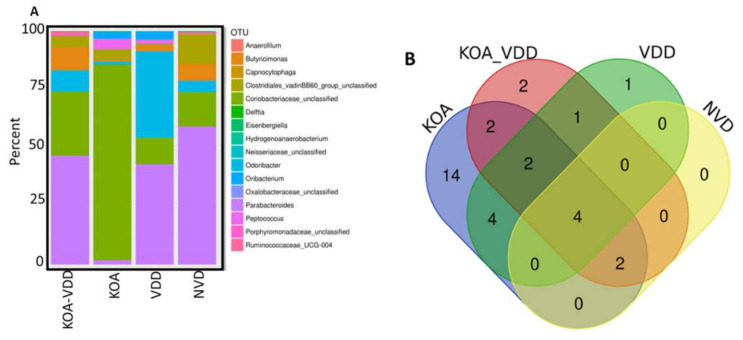

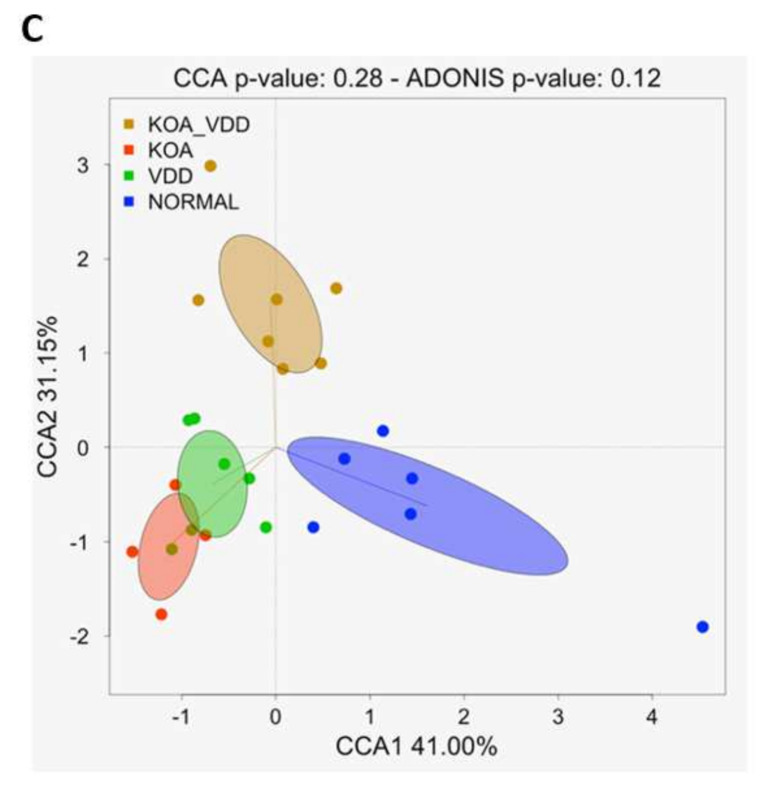
Gut microbiome profile: (**A**) Operational Taxonomic Units (OTU) abundance was determined in stool samples collected across 4 study groups depicted in the bar chart; (**B**) Venn diagram with filtered OTUs excluding counts less than 50 OTUs; (**C**) canonical correspondence analysis (CCA) was performed to study the beta diversity between stool microbiome in Knee osteoarthritis (KOA)_vitamin D deficiency (VDD), KOA, VDD and Normal Participants.

We determined the gut microbiome composition through the analysis of the faecal microbiome. To identify bacterial taxa, we sequenced the V3–V4 hypervariable regions of the bacterial 16S rRNA gene using an Illumina MiSeq system. After quality control and filtering, ~3,401,904 high-quality sequences with an average length of 600 bp were recovered for further analysis, with an average of ~141,746 reads per sample (ranging from 68,540 to 250,424 reads) (Table S1). The rarefaction curves obtained for each sample reached a plateau, indicating sufficient sequencing coverage depth (Figure S1). The mapping of the 16S rDNA sequencing reads against the Silva 16S sequence database (v128) using RDP classifier led to the identification of 447 distinct OTUs. The gut microbiome composition of 24 participants was then schematically presented in Figure 1A. At the phylum level, the dominant phyla were Firmicutes (59.4%) and Proteobacteria (18.6%), followed by Tenericutes (11%) and Actinobacteria (6.5%) and Bacteroidetes (2.7%). Using the Venn diagram, we observed that all four groups shared 217 OTUs, whereas 32, 39, 33 and 23 OTUs were specific to the KOA, KOA_VDD, VDD and NVD groups, respectively (Figure S2). However, when OTUs with counts less than 50 were removed, all four groups shared only four OTUs, whereas 14, 2, 1, and 0 OTUs were specific to the KOA (Table S2), KOA_VDD, VDD and normal groups, respectively (Figure 1B). We also evaluated the overall differences (Table S3) in beta diversity between the faecal microbiome of KOA_VDD, KOA, VDD and Normal Participants using canonical correspondence analysis (CCA), based on Bray–Curtis distances (Figure 1C). All study groups emerge to cluster differently, although the ADONIS test does not explain this difference statistically (*p* = 0.12).

## 7. Knee Osteoarthritis-Associated Dysbiosis

**Figure 2:**
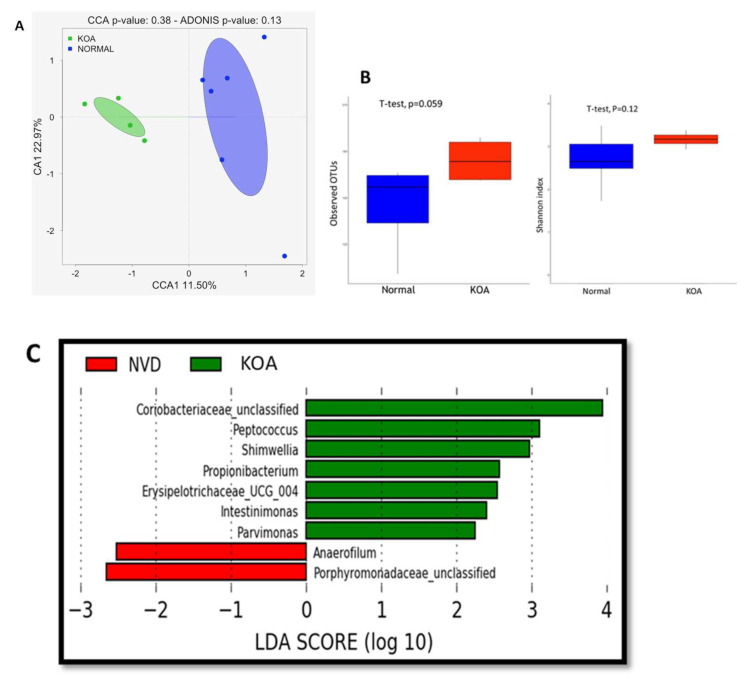
Knee osteoarthritis-associated dysbiosis: (**A**) canonical correspondence analysis (CCA) was performed to study the beta diversity between stool microbiome in KOA and Normal participants; (**B**) Alpha-diversity using the observed OTU and Shannon index based on Bray–Curtis distances; (**C**) A histogram of the log 10 transformed Linear discriminant analysis (LDA) scores was computed for features that showed differential abundance between healthy subjects and KOA patients.

To assess the effect of Knee osteoarthritis disease on the gut microbiome, we compared the gut microbiome composition of individuals with normal vitamin D status, diagnosed with Knee osteoarthritis disease (KOA) and those considered as “healthy” (NVD). We first evaluated the overall differences in beta diversity between faecal microbiome samples of both groups using canonical correspondence analysis (CCA), based on Bray–Curtis distances (Figure 2A). Although the ADONIS test does not explain this difference (*p* = 0.13) statistically, the two groups appear to cluster differently.

We estimated α-diversity using the observed OTU and Shannon index (using the Shannon index measuring how evenly OTUs are distributed in a sample) in both groups (Figure 2B). KOA samples had overall higher bacterial diversity compared to those of healthy participants (observed OTU index, 174.2 ± 14.8 vs. 145.7 ± 26.2; and Shannon index, 3.2 ± 0.19 vs. 2.7 ± 0.6), although these differences are not statistically significant.

To investigate differentially abundant taxa between both groups, we then performed LEfSe analysis to compare the abundance of bacterial taxa in KOA patients and healthy subjects. A histogram of the Linear Discriminate Analysis scores was computed for features that showed differential abundance between healthy (NVD) subjects and KOA patients (Figure 2C). The Linear discriminant analysis (LDA) scores indicated that the relative abundances of Peptococcus, Shimwellia, Propionibacterium, Intestinimonas and Pavimonas were more enriched in patients with KOA patients than in healthy (NVD) subjects. The most differentially abundant bacterial taxon in patients with KOA was Peptococcus, and for the NVD group, it was Anaerofilum with a three-fold difference in LDA score.

### 7.1. Knee Osteoarthritis-Associated Dysbiosis in Patients with Vitamin D Deficiency

**Figure 3:**
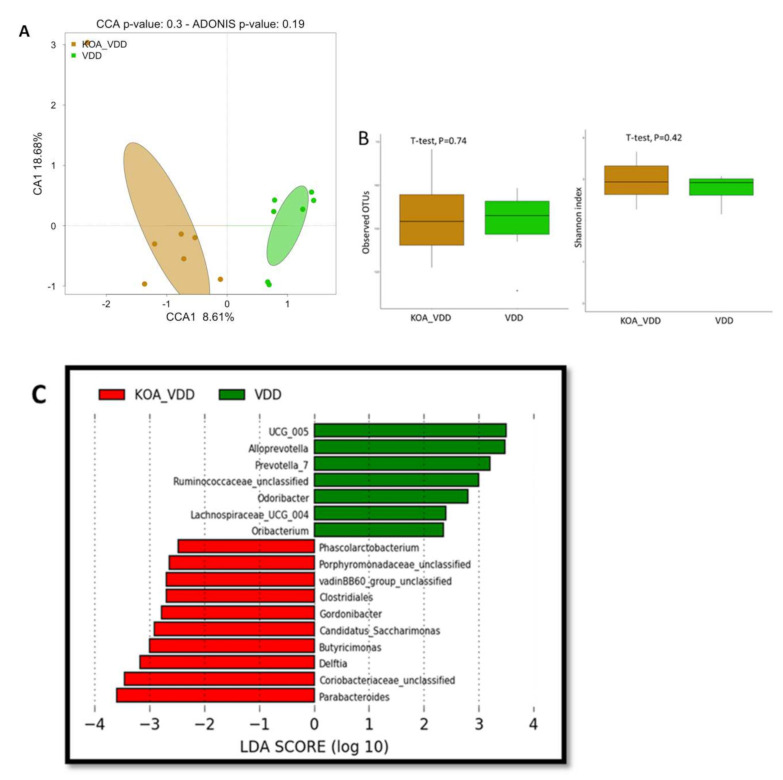
Knee osteoarthritis-associated dysbiosis in patients with D vitamin deficiency: (**A**) canonical correspondence analysis (CCA) was performed to study the beta diversity between stool microbiome in KOA_VDD and VDD patients; (**B**) Alpha-diversity using the observed OTU and Shannon index based on Bray–Curtis distances and (**C**) Histogram of the log 10 transformed LDA scores were computed for features that showed differential abundance between KOA_VDD and VDD patients.

To assess the effect of vitamin D deficiency on Knee osteoarthritis disease on the gut microbiome, we compared the gut microbiome composition of individuals with Knee osteoarthritis patient with vitamin D deficiency (KOA_VDD) and those with vitamin D deficient (VDD). We first evaluated the overall differences in beta diversity between faecal microbiome samples of both groups using canonical correspondence analysis (CCA), based on Bray–Curtis distances (Figure 3A). Although the ADONIS test does not explain this difference (*p* = 0.19) statistically, the two groups appear to cluster differently.

We estimated α-diversity using the observed OTU and Shannon index (using the Shannon index measuring how evenly OTUs are distributed in a sample) in both groups (Figure 3B), and they were not statistically significant (observed OTU index, *p* = 0.7; and Shannon index, *p* = 0.42).

However, when LEfSe analysis was performed between KOA_VDD and VDD, the gut microbiome of KOA_VDD was found enriched with Phascolarctobacterium, Gordonibacter, Delftia, Parabacteroides, Candidatus-saccharimoanas and Butyricimonas. In contrast, the gut microbiome in VDD was formed predominantly by Alloprevotella, Odoribacter and Oribacterium (Figure 3C).

### 7.2. Vitamin D Deficiency Impacts Knee Osteoarthritis-Associated Dysbiosis

**Figure 4:**
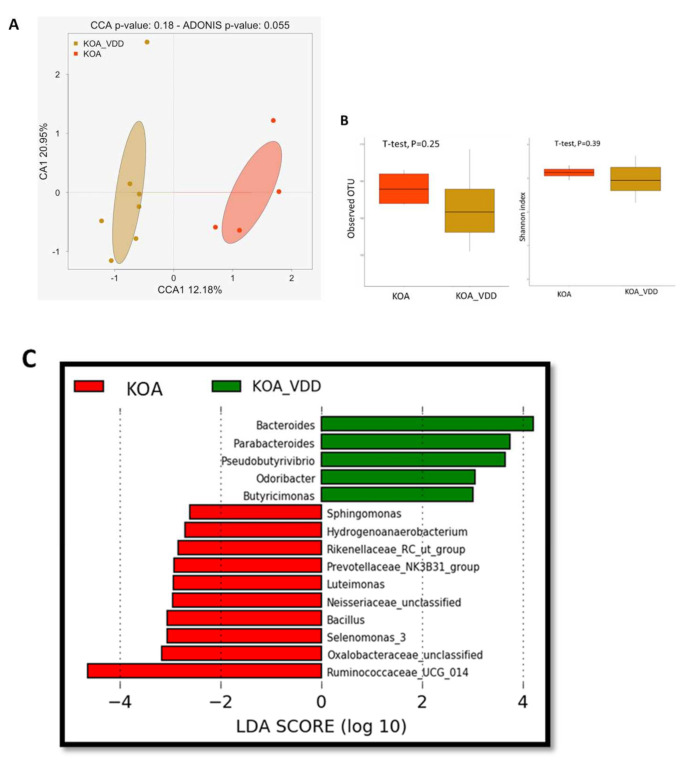
Effect of vitamin D deficiency on Knee osteoarthritis-associated dysbiosis: (**A**) canonical correspondence analysis (CCA) was performed to study the beta diversity between stool microbiome in KOA_VDD and KOA patients; (**B**) Alpha-diversity using the observed OTU and Shannon index based on Bray–Curtis distances; (**C**) A histogram of the log 10 transformed LDA scores was computed for features that showed differential abundance between KOA_VDD and KOA_NVD patients.

We assessed the effect of the vitamin D deficiency on the gut microbiome of KOA patients by comparing the gut microbiome of KOA patients with and without vitamin D deficiency (KOA vs KOA_VDD). Although the CCA analysis presents two distinct clusters representing the KOA and KOA_VDD groups, this difference is not significantly different (Adonis, *p* = 0.055) (Figure 4A). We also observed a decreased alpha diversity in the KOA_VDD group compared to the KOA (observed OTU index, 158.3 ± 28.4 vs 174.2 ± 14.8; and Shannon index, 3.0 ± 0.5 vs 3.2 ± 0.19), but this difference was not statistically different (Figure 4B). However, as revealed by the LEfSe analysis, the gut microbiome of KOA_VDD is enriched by bacterial taxa, including Bacteroides and Parabacteroides Pseudobutyrivibrio, Odoribacter and Butyricimonas. On the other hand, the KOA microbiome was characterized by OTUs, including Sphingomonas, Hydrogenoanaerobacterium, Rickenellaceae, Luteimonas, Selenomonas, Oxalobacteraceae, Ruminococcaceae and Neisseriaceae (Figure 4C).

### 7.3. Vitamin D Deficiency Affects the Gut Bacterial Communities

**Figure 5:**
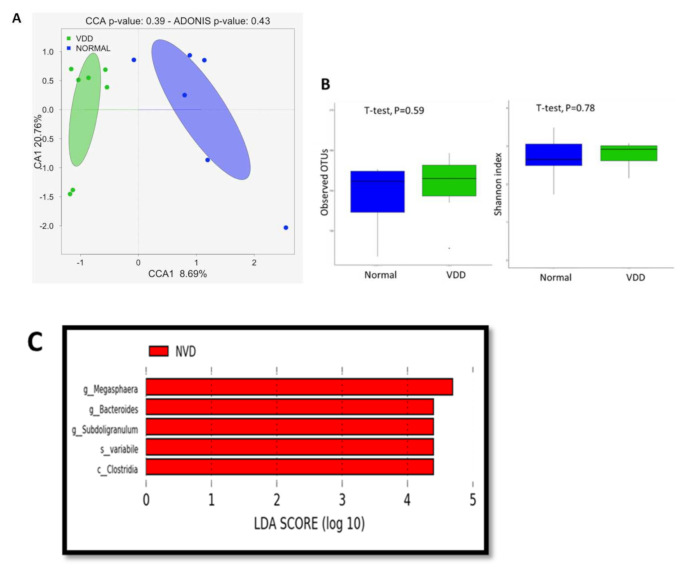
Vitamin D deficiency affects the gut bacterial communities: (**A**) canonical correspondence analysis (CCA) was performed to study the beta diversity between stool microbiome in VDD and NVD participants; (**B**) Alpha-diversity using the observed OTU and Shannon index based on Bray–Curtis distance–s and (**C**) Histogram of the log 10 transformed LDA scores were computed for features that showed differential abundance between VDD and NVD participants.

To investigate the effect of the vitamin D deficiency on the gut microbiome composition, we analyzed the gut microbiome composition of participants with levels of vitamin D considered as deficient (<30 ng/mL, VDD group) and normal (>30 ng/mL, NVD group). Globally, as shown, the CCA analyzed (Figure 5A) the VDD and NVD groups clustered separately, displaying an effect of the vitamin D deficiency on the gut microbiome. However, the ADONIS test did not confirm it (ADONIS, *p* = 0.43). We observed that the vitamin D deficiency tended to increase the alpha diversity (Figure 5B) (observed OTU index, 153.4 ± 24.2 vs. 145.7 ± 26.2; and Shannon index, 2.8 ± 0.3 vs. 2.7 ± 0.6), although it is not statistically significant. Nevertheless, vitamin D deficiency significantly affects the abundance of some taxa, as the LEfSe analysis shows (Figure 5C). The deficiency of vitamin D was associated with decreased abundance of five OTUs (LDA score [log10] > 4), including Megasphaera (Genus level), Bacteroides (Genus level), Subdologranulum (Genus level), Paradoxostoma variabile (Species-level) and Clostridia (Class level).

On the other hand, when LEfSe Analysis was performed on all four study groups (Figure S3), the distinct gut microbiome patterns were identified. The distinct OTUs for KOA were Peptococcus, Delftia and Oribacterium, while it was Gordonibacter, Butyricimonas and Parabacteroides for KOA_VDD. While Pseudobutyrivibrio and Odoribacter are specific for VDD and Normal group (NVD) was enriched with Faecalibacterium and Anaerofilum. These distinctions are significant and show a strong association between vitamin D status, microbiome and KOA.

## 8. Discussion

This study reveals for the first time interaction of vitamin D and the KOA status on the gut microbiome. In a well-defined KOA phenotype, we observed an effect of the vitamin D status on the gut microbiome in KOA patients. Significant enrichment of specific OTUs was observed between KOA patients with and without vitamin D deficiency.

Previously, Jackson et al. 2018 reported that the abundance of specific gut microbes (*Lentispherea*) was negatively associated with OA and rheumatoid arthritis [34]. As expected, the gut microbiome of KOA patients included in our study (KOA) was characterized by a dysbiosis compared with that of healthy individuals (NVD groups). It is suggested that the gut microbiome interacts with risk factors of OA and modulates the disease process. Local and systemic inflammation have an association with KOA. The release of proinflammatory mediators (TNF-α, IL-6) is augmented by numerous mechanisms such as epigenetic alterations, mitochondrial dysregulation or cellular senescence. Previous literature showed increased proinflammatory anaerobes and decreased anti-inflammatory microbes (i.e., *Faecalibacterium prauznitzii*) in the gut with ageing. Besides, our study showed that *Faecalibacterium* is enriched in the NVD group compared to the KOA_VDD group, suggesting that the KOA-associated microbiome could participate in the inflammation process and the degeneration process in the joint.

A previous study based on large-scale population-cohorts of Caucasian adults found a microbiome-wide association with knee WOMAC pain and *Streptococcus* spp. [34]. However, they have not considered vitamin D status in their study, although they considered individuals’ BMI status in their analysis.

To our knowledge, this is the first clinical observation study exploring the association between vitamin D status and gut microbiome in KOA patients. Our study observed the effect of vitamin deficiency on the gut microbiome adequately. Compared to healthy individuals (NVD), Pseudobutyrivibrio and Odoribacter are retrieved specifically in VDD patients. Interestingly we also observed an effect of the vitamin status on the KOA-associated dysbiosis (Figures S4–S6). The gut microbiome of KOA patients with deficient vitamin D (KOA_VDD) was characterized by an increased abundance of Parabacteroides, Butyricimonas, Gordonibacter, while Intestimonas, Delftia, Peptococcus were specific for KOA. These results suggest that vitamin deficiency shapes the KOA linked gut microbiome. In effect, distinct signatures were observed when KOA (KOA) was compared with healthy individuals and vitamin deficient KOA (KOA_VDD) with vitamin-deficient patients (VDD) (Figure S5). This reveals that vitamin deficiency affects the gut microbiome, but these alterations are dependent on the KOA status of individuals. It was probable that the unfavourable effects of vitamin D deficiency in the KOA disease could be mediated in part through the gut microbiome. Conversely, vitamin D supplementation likely exerts a beneficial role on the KOA-altered gut microbiome, thus modulating the disease process of OA. Nevertheless, the determination of the role of this interaction needs to be determined in the future.

In support of this observation, the Phylogenetic Investigation of Communities by Reconstruction of Unobserved States (PICRUSt) analysis of our study revealed that the gut microbiome involved in lipopolysaccharide biosynthesis and lipopolysaccharide biosynthesis protein is seen in the KOA_VDD group (Figure S7). Along with these functions, we observed that cell mobility, secretions, cellular antigens, transcription related proteins and glycan synthesis and metabolism are also controlled by the gut microbiome found in KOA_VDD patients. On the other hand, amino acid metabolism (taurine and hypotaurine metabolism, cyanoaminoacid metabolism), glycolysis, gluconeogenesis, pyruvate metabolism and phosphatidylinositol signalling system are controlled by the gut microbiome of the KOA group. These observations indicate that vitamin D probably controls the functionality of the gut microbiome responsible for the production of lipopolysaccharide and cell mobility, secretion, and cellular antigen expression.

Vitamin D is known to induce E-cadherin expression and improve the epithelial cell junction in the gut. In contrast, vitamin D deficiency causes the breakdown of the junction resulting in a leaky gut and favouring the passage of bacterial components and product from the bacterial metabolism. In this way, KOA patients with a deficiency in D vitamin could have increased permeability of the gut barrier. Bacterial peptidoglycans were detected in the synovial fluid of patients with reactive arthritis and OA, suggesting that these antigens result in inflammation of the joints. In a study, rats injected with *Propionibacterium acnes* into the shoulder joint developed erosive arthritis, showing microorganisms and bacterial components in the inflammatory process of the joint. The common bacterial pathogens detected in joint inflammation correspond to bacteria retrieved in the gut microbiome, such as *Pseudomonas* sp., *Shigella* sp. and *Escherichia coli* species.

Osteoarthritis requires lifelong treatment either for a cure or for arresting the progression of the disease. Because of its plasticity, the gut microbiome is an exciting target for preventive or therapeutic interventions. The data generated by our study show promising leads for effectively relieving indications of KOA. The microbes observed in VDD without KOA (Lachnospiraceae, Xylanivorans, pseudobutyrivibrio, Dialister) may offer protection from KOA, despite a deficiency of vitamin D as these patients did not develop KOA. Specifically, identifying bacterial species belonging to these taxa could lead to identifying newer probiotics with potential benefits for the KOA. As shown in our study’s PICRUSt analysis (Figure S7), the modulation of metabolic process, cellular mobility, cellular secretion, and antigen expression with LPS formation are predominant actions on the KOA-associated gut microbiome. Earlier studies on oral ingestion of *Lactobacillus casei* (2 × 10^10^ CFU, 500 mg/kg of body weight) with type II collagen (250 mg/ kg body weight) and glucosamine (250 mg/kg body weight) reduced pain, cartilage destruction and pannus formation compared to Glucosamine or *L. Casei* alone in rat RA model_ENREF_15. *L casei* is a probiotic that suppresses the proinflammatory cytokines (IL-1b, IL-6, etc.) and increases the anti-inflammatory cytokines (IL-10 and IL-4) in the rat OA model. To date, there are no human studies that have investigated the role of probiotics in osteoarthritis.

These findings are based on the analysis of a few individuals, and they need to be confirmed on a larger cohort. However, the patient groups included in our study were homogenous in terms of dietary intake, smoking, alcohol intake, oral medication usage, physical activity, sun exposure and BMI, which confirms that the observed interaction between KOA and vitamin D status is not produced by other confounding factors and strengthens our data.

To conclude, the gut microbiome in knee osteoarthritis patients with or without vitamin D deficiency presents different bacterial abundances. Form these data; we suggest that the impact of vitamin D deficiency in the KOA disease could be mediated through the gut microbiome. These observations recommend a larger vitamin D interventional study in KOA patients to confirm the beneficial effects of resetting the observed dysbiosis on the progression of KOA.

## Figures and Tables

**Table 1:** V3–V4 region-specific primers for 16s rRNA gene amplification.

V3-4F	AATGATACGGCGACCACCGAGATCTACACTCTTTCCCTACACGACGCTCTTCCGATCTNNNNACTCCTACGGGAGGCAGCAG
V3-4R	CAAGCAGAAGACGGCATACGAGATXXXXXXGTGACTGGAGTTCAGACGTGTGCTCTTCCGATCTGGACTACHVGGGTWTCTAAT

X: Index Barcode; N: Degenerative bases; Bases in Capitals: V3–V4 specific primer sequence.

**Table 2:** Clinico-Demographic profile.

Characteristics	KOA_VDD (*n* = 7)	KOA (*n* = 4)	VDD (*n* = 7)	Normal (NVD) (*n* = 6)
Age (Years ± SD)	52 ± 7.2	50 ± 9.70	44 ± 8.1	37.7 ± 12.7
Gender (Male/Female)	1/6	1/3	7/0	7/0
Clinical Presentation				
Painful Knee	++	++	NA	NA
WOMAC Score (Score ± SD)	57.6 ± 7.2	54.3 ± 10.7	90 ± 2.2	93 ± 0.4
Kellgren-Lawrence grade (Grade ± SD)	2.3 ± 0.9	3.5 ± 0.6	NA	NA
Demography				
Vitamin D ng/mL (Levels ± SD)	21.2 ± 3.5	35.8 ± 4.2	19.1 ± 4.3	42 ± 9.8
Hemoglobin g/dL (levels ± SD)	12.8 ± 1.7	12.7 ± 1.9	13.1 ± 2.1	13.1 ± 1.8
BMI	28.2 ± 2.7	27.5 ± 2.8	28.9 ± 2.6	22.9 ± 2.6
Physical activity	Mild	Mild to	Moderate	Active
Socio-economic score (Score ± SD)	13.7± 4.7	12± 2.4	20± 5.2	14.1± 2.1

KOA_VDD—Knee osteoarthritis with Vitamin D Deficiency, KOA—Knee osteoarthritis, VDD—Vitamin D Deficiency, NVD—Normal Vitamin D Status. ++ is the Pain Score.

## Data Availability

Sequence data are available in the NCBI Nucleotide Archive under SRA: SRP 299187 and BioProject: PRJNA 687346.

## Supplementary Materials

The following are available online at https://www.mdpi.com/article/10.3390/nu13041272/s1, Table S1: Sequence data quality parameters and reads per sample, Table S2: OTUs that are predominant in various study groups. This classification is based on excluding OTUs with counts less than 50 in each of the groups, Table S3: OTUs with Differential abundance between groups, Figure S1: Overall quality of data, Figure S2: Venn diagram with excluding zero counts to OTUs in each of the groups, Figures S3–S6: LEfSe analysis, Figure S7: PiCRUSt analysis computed for features that showed differential abundance between all study groups.
