# Supplementary material for: Association of Gut Microbiome and Vitamin D Deficiency in Knee Osteoarthritis Patients: A Pilot Study
Source: Nutrients. 2021 Apr 13;13(4):1272. doi: 10.3390/nu13041272 (PMC8069973; doi:10.3390/nu13041272)
Supplement: Supplementary file 1 [file nutrients-13-01272-s001.pdf]

Supplementary Information

Table S1: Sequence data quality parameters and reads per sample:

Sample	Number of reads	%GC	% >Q20	% >Q30
Sample_01	111212	53.5	99	97
Sample_02	68540	54	96	95
Sample_03	124272	54.5	98	96
Sample_04	80876	54	98	94
Sample_05	115504	54	98	96
Sample_06	127260	54.5	99	95
Sample_07	87790	52.5	99	96
Sample_08	165130	51.5	99	97
Sample_09	184340	53.5	99	95
Sample_10	92254	54	99	96
Sample_11	108494	54	98	96
Sample_12	185588	52	99	97
Sample_13	98408	54	99	95
Sample_14	158590	53	98	96
Sample_15	192958	52	99	97
Sample_16	156140	53	99	96
Sample_17	190170	55.5	99	96
Sample_18	131700	52.5	99	96
Sample_19	250424	52.5	99	96
Sample_20	97374	53	99	97
Sample_21	182782	53.5	99	95
Sample_22	175410	53	99	97
Sample_26	136668	52.5	99	96
Sample_27	180020	51.5	99	96

Table S2: OTUs that are predominant in various study groups. This classification is based on excluding OTUs with counts less than 50 in each of the groups.

**KOA KOA_VDD NVD VDD**	4	Bifidobacterium
		Faecalibacterium
		Collinsella
		Blautia
**KOA KOA_VDD VDD**	2	Prevotella_9
		Lachnospiraceae_unclassified
**KOA KOA_VDD NVD**	2	Bacteroides
		Subdoligranulum
**KOA KOA_VDD**	2	Dorea
		Catenibacterium
**KOA VDD**	4	Alloprevotella
		Dialister
		[Eubacterium]_coprostanoligenes_group
		Ruminococcaceae_UCG-002
**KOA_VDD VDD**	1	Pseudobutyrivibrio
**KOA**	14	Coriobacteriaceae_unclassified
		Streptococcus
		Olsenella
		Coprococcus_2
		Ruminococcaceae_UCG-005
		Anaerotruncus
		Christensenellaceae_R-7_group
		Prevotellaceae_unclassified
		Peptostreptococcaceae_unclassified
		Lachnospiraceae_NK4A136_group
		Ruminococcus_2
		Ruminococcaceae_UCG-014
		Ruminococcus_1
		Senegalimassilia
**KOA_VDD**	2	Parabacteroides
		Escherichia-Shigella
**VDD**	1	Lachnospiraceae_UCG-005

Table S3: OTUs with Differential abundance between groups.

OTU	P value	Adjusted P value*
Butyricimonas	0.011	0.730
Parabacteroides	0.014	0.730
Oxalobacteraceae_unclassified	0.015	0.730
Neisseriaceae_unclassified	0.015	0.730
Hydrogenoanaerobacterium	0.015	0.730
Coriobacteriaceae_unclassified	0.016	0.730
Anaerofilum	0.016	0.730
Delftia	0.021	0.730
Porphyromonadaceae_unclassified	0.021	0.730
Odoribacter	0.025	0.730
Oribacterium	0.037	0.730
Clostridiales_vadinBB60_group_unclassified	0.044	0.730
Peptococcus	0.047	0.730
Ruminococcaceae_UCG-004	0.047	0.730
Capnocytophaga	0.047	0.730
Eisenbergiella	0.047	0.730

*Benjamini Hochberg adjusted.

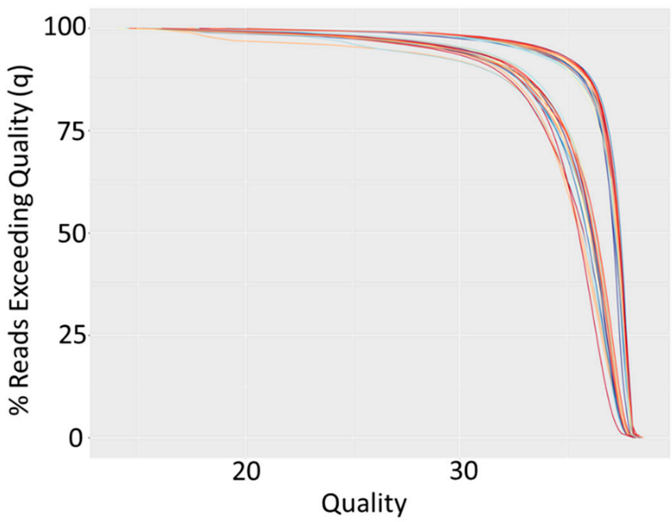

Figure S1: Overall quality of data: Fast QC and R qc were used to check the quality parameters of sequence data like, base call quality distribution Figure S1, % bases above Q30, GC% (Table S1), Sequencing adapter contamination etc.

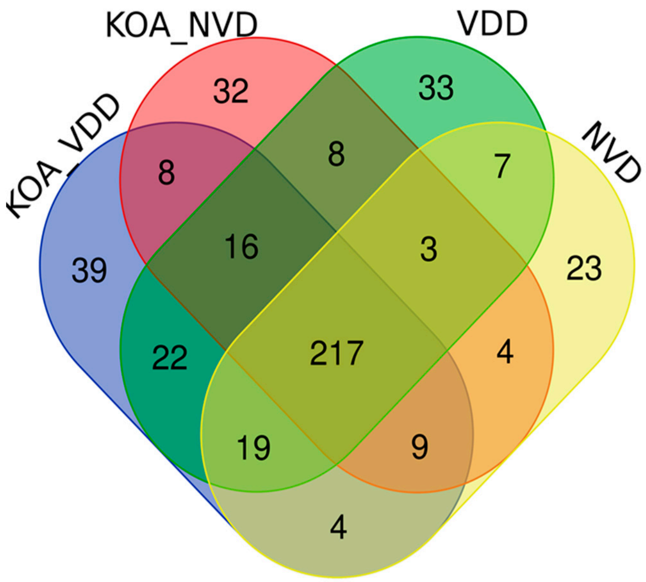

Figure S2. Venn diagram with excluding zero counts to OTUs in each of the groups.

LEfSe (Linear discriminant analysis effect size)

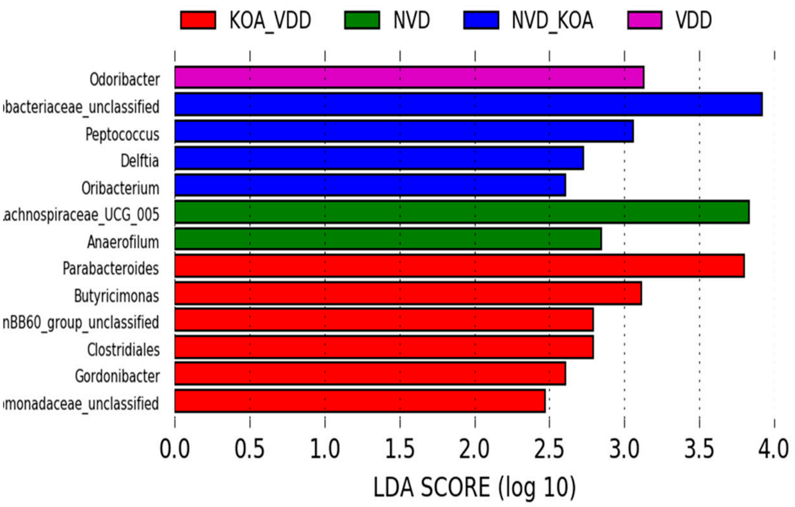

Figure S3: Linear discriminant analysis (LDA) scores computed for features that showed differential abundance between study subjects.

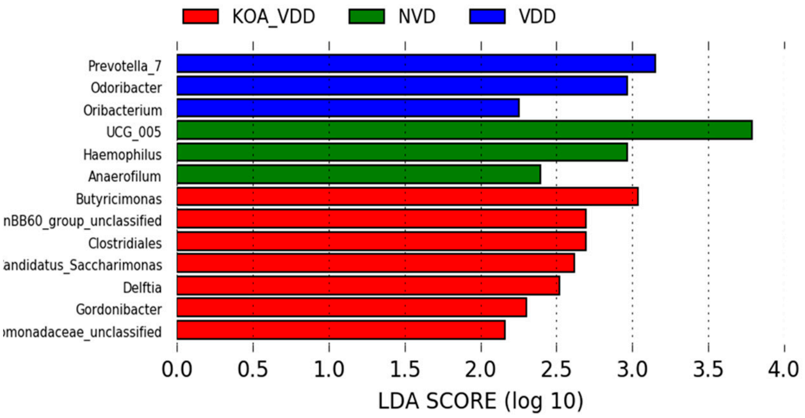

Figure S4: Linear discriminant analysis (LDA) scores computed for features that showed differential abundance between KOA_VDD, NVD an VDD.

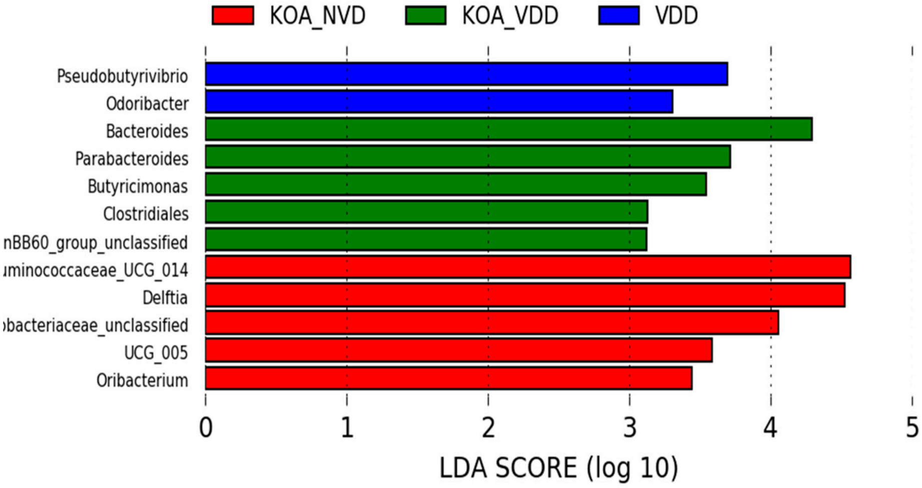

Figure S5: Linear discriminant analysis (LDA) scores computed for features that showed differential abundance between KOA_NVD, KOA_VDD an VDD.

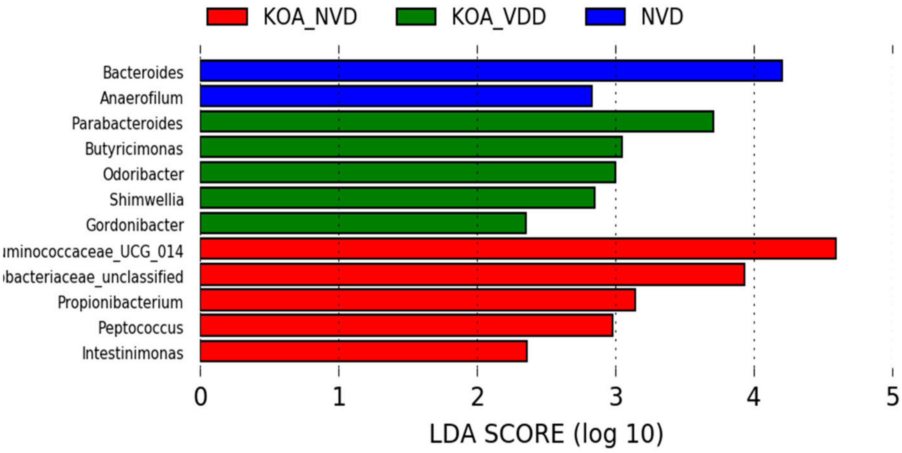

Figure S6: Linear discriminant analysis (LDA) scores computed for features that showed differential abundance between KOA_VDD, KOA_NVD an NVD.

Phylogenetic Investigation of Communities by Reconstruction of Unobserved States (PiCRUSt)

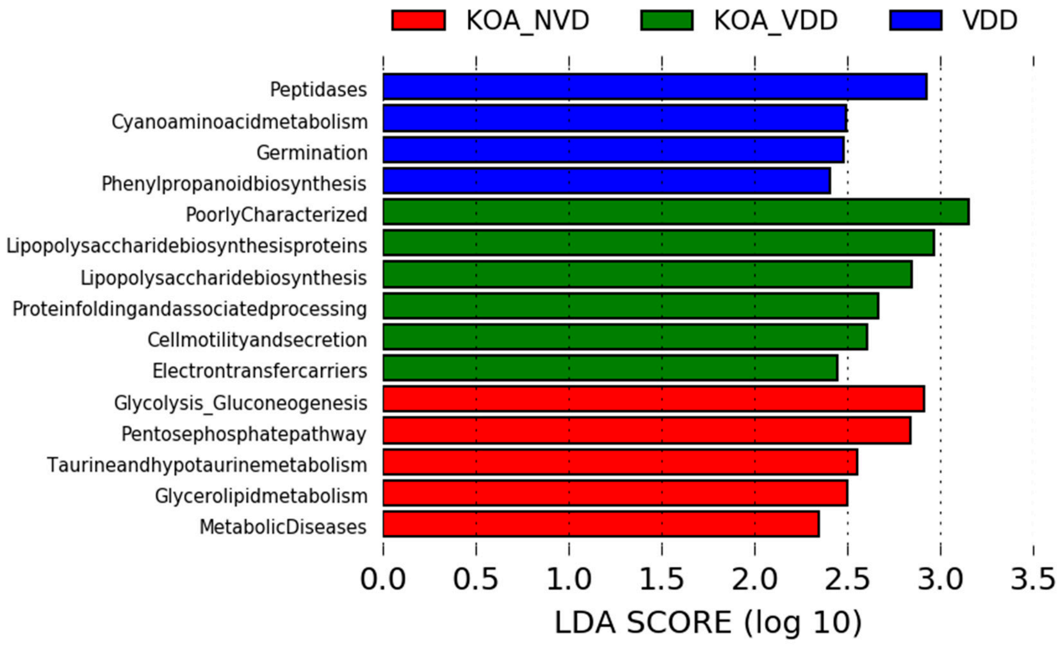

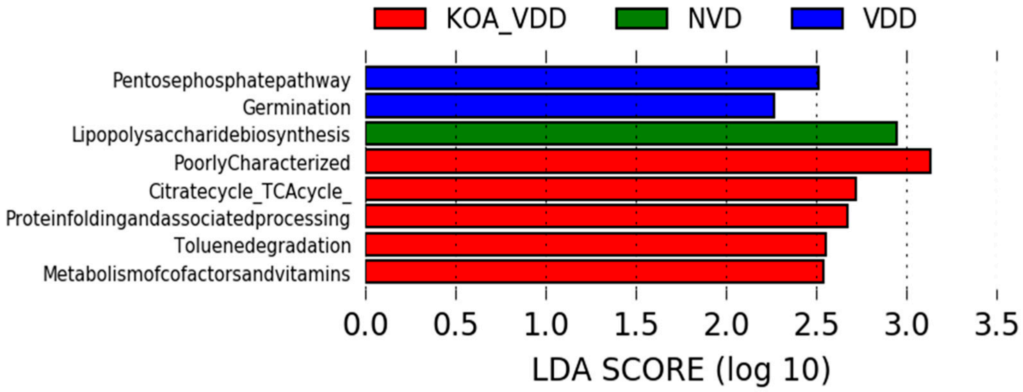

Figure S7: PiCRUSt analysis computed for features that showed differential abundance between all study groups.
